# Serum bile salt-stimulated lipase levels associates with systemic inflammation and declines with effect of treatment in juvenile idiopathic arthritis

**DOI:** 10.1186/s12969-026-01191-x

**Published:** 2026-02-12

**Authors:** Lillemor Berntson, Olle Hernell, Kajsa Linde, Eva-Lotta Andersson, Susanne Lindquist

**Affiliations:** 1 Department of Women’s and Children’s Health, Uppsala University, Uppsala, Sweden; 2 Lipum AB, Umeå, Sweden; 3 Department of Clinical Sciences, Pediatrics, Umeå University, Umeå, Sweden

**Keywords:** Juvenile idiopathic arthritis, Biomarker, Bile salt-stimulated lipase

## Abstract

**Background:**

Juvenile idiopathic arthritis (JIA) is among the most common autoimmune diseases in children, yet its pathophysiology remains incompletely understood. Reliable biomarkers are needed to assess disease activity and guide therapy. Bile salt-stimulated lipase (BSSL), originally identified as a lipolytic enzyme, has recently been implicated in inflammatory conditions such as rheumatoid arthritis (RA) and psoriatic arthritis (PsA) in adults. Elevated serum BSSL levels correlate with markers of inflammation and decrease following positive response to anti-inflammatory treatments, suggesting potential utility as both a biomarker and a therapeutic target.

**Methods:**

Serum BSSL concentrations were measured in children with JIA and compared with healthy controls. Correlations between BSSL and inflammatory markers (S100A8/A9, HNL), blood cell counts (leukocytes, neutrophils, platelets), and clinical disease-activity indices (ESR, JADAS27, cJADAS27, CHAQ) were evaluated.

**Results:**

Children with JIA had significantly higher serum BSSL concentrations than healthy controls, and levels decreased following medical treatment. BSSL correlated positively with platelets, ESR, and S100A8/A9, but not with JADAS27.

**Conclusion:**

Serum BSSL tracks systemic inflammatory activity and decreases with clinical improvement in JIA, supporting its further evaluation as a complementary inflammatory biomarker and potential pharmacodynamic read out in pediatric inflammatory arthritis.

## Background

Juvenile idiopathic arthritis (JIA) is one of the most common autoimmune diseases in children, encompassing seven categories of inflammatory articular disorders with onset before 16 years of age and a duration of at least 6 weeks [1]. Although several JIA categories have adult counterparts, the most prevalent subtype, oligoarticular JIA, is unique to children. In contrast, polyarticular rheumatoid factor (RF)-positive JIA closely resembles adult rheumatoid arthritis (RA), but accounts for only 3–5% in children [2].

The pathogenesis of JIA involves both genetic and environmental factors [3]. Early research emphasized dysregulation within the adaptive immune system, particularly T and B lymphocytes, as crucial for the development of JIA [3]. However, emerging evidence also highlights a critical role for innate immunity, especially neutrophil granulocytes, in driving the inflammatory process. One of the best-characterized neutrophil-derived proteins, S100A8/A9, also known as calprotectin or MRP8/14, reflects disease activity in several JIA categories [4, 5]. S100A8/A9 constitutes approximately half of the cytosolic protein content in neutrophils and is released during cell turnover and sequestration [6]. Moreover, several genes are differentially expressed in neutrophils from patients with polyarticular JIA compared with healthy controls, and the abnormal pattern of neutrophil gene expression persists despite treatment and even after clinical improvment. These findings indicate that neutrophils in JIA remain chronically activated, underscoring the pivotal role of the innate immune system in JIA pathophysiology [7]. Another neutrophil-related protein, the dimeric form of human neutrophil lipocalin (HNL), also referred to as neutrophil gelatinase-associated lipocalin (NGAL), has been studied in JIA [8]. While HNL is secreted upon neutrophil priming, its correlation with disease activity appears limited. These findings collectively suggest that additional factors, beyond the well-studied S100A8/A9 and HNL, may underlie the persistent neutrophil-driven inflammation in JIA.

Bile salt-stimulated lipase (BSSL; EC 3.1.1.13), also known as carboxyl ester lipase (CEL), bile salt-dependent lipase (BSDL), bile salt-activated lipase (BAL), or carboxyl ester hydrolase (CEH), has emerged as a novel player in inflammatory processes. Initially, BSSL was recognized as a lipolytic enzyme secreted from the exocrine pancreas and in some species, notably humans, also from the lactating mammary gland as a constituent of the milk [9]. BSSL is a multifunctional protein that contributes to efficient digestion and absorption of dietary fat, particularly in breastfed infants [10, 11], and has also been linked to the protective effect of mother’s milk against viral infections [12–14]. The presence of BSSL is not confined to the gastrointestinal tract; it is also found in peripheral blood and its concentration is elevated in some pathological conditions, e.g., acute pancreatitis [15, 16]. BSSL levels are also higher in patients with rheumatoid arthritis (RA) and psoriactic arthritis (PsA), correlate with inflammation markers, and can be modulated by anti-inflammatory treatments [17]. Multiple cell types, including macrophages [18], endothelial cells [19], and platelets [20], may produce or store BSSL, supporting broader physiological and pathological functions beyond lipid metabolism.

Consistent with elevated circulating BSSL levels in RA and PsA patients [17], studies in rodent models demonstrate that BSSL is crucial for the development of inflammatory arthritis. Conventional BSSL knockout mice show markedly reduced susceptibility to collagen-induced arthritis, and treatment with antibodies directed against BSSL effectively mitigates experimental arthritis in both mice and rats [21]. In vitro, BSSL promotes the migration of human leukocytes, particularly CD14 + monocytes [17]. Collectively, these findings suggest that BSSL functions as a pro-inflammatory mediator of potential clinical relevance.

Despite these insights, the possible role of BSSL in JIA remains unexplored. Based on evidence linking BSSL to inflammatory pathways in adult RA, we hypothesized that BSSL may similarly contribute to the pathophysiology of JIA. In the present study, we quantified serum BSSL concentrations in children with JIA and compared them with healthy controls. We further evaluated correlations between BSSL levels and established inflammatory markers (S100A8/A9, HNL), hematologic variables (leukocytes, neutrophils, platelets), and clinical disease-activity indices (ESR, JADAS27, cJADAS27, CHAQ). The overarching aim was to explore whether BSSL could serve as a novel biomarker of systemic inflammation or treatment response, and potentially represent a future therapeutic target, in pediatric inflammatory arthritis.

## Materials and methods

### Study population

#### Patients with JIA

A total of 102 patients with JIA were recruited from the unit of Pediatric Rheumatology, Uppsala University Hospital, Uppsala, Sweden. All patients met the International League of Association for Rheumatology (ILAR) criteria for JIA [1]. At inclusion, the majority of patients (*n* = 93, 91.2%) were either newly diagnosed or experiencing a disease flare and were on no medical treatment. Nine (8.8%) participants were receiving methotrexate or low-dose prednisolone (Table 1).

**Table 1:** Demographic data for 102 children with juvenile idiopathic arthritis

	Total cohort *N* = 102	Children providing samples at follow-up *N* = 12	Healthy children *N* = 20
Age at inclusion, Md (IQR)	11.9 (5.2–15.0)		10.3 (7.3–13.9)*
Age at onset, Md (IQR)	10.2 (2.9–13.5)		
Sex (F/M), n (%)	67/35 (65.7/34.3)		11/9 (55.0/45.0)**
Disease duration between sampling, year, Md (IQR)		1.2 (0.6–1.7)	
JADAS27 at baseline, *n* = 12, Md (IQR)		13.5 (10.9–20.7)	
JADAS27 at follow-up, *n* = 11, Md (IQR)		0.0 (0.0–4.2)	
*Disease activity state*			
At onset, no medication During flare, no medical treatment During flare, on medical treatment	59 34 9		
**ILAR categories**,** course type**,** n (%)**			
Oligoarticular persistent	57 (55.8)		
Polyarticular RF negative	15 (14.7)		
Enthesitis-related arthritis	15 (14.7)		
Polyarticular RF positive	5 (4.9)		
Juvenile psoriatic arthritis	7 (6.8)		
Systemic	2 (2.0)		
Oligoarticular extended	1 (1.0)		
**Medical treatment**,** n (%)**			
No medical treatment	93 (91.2)	3 (25.0)	
Methotrexate	6 (5.8)	3 (25.0)	
Prednisolone (0.06/0.67/5.0 mg/kg/d)	3 (3.0)		
TNF-inhibitor TNF-inhibitor + methotrexate		3 (25.0) 3 (25.0)	

Abbreviations: Md = median, IQR = interquartile range, F = female, M = male, JADAS27 = juvenile arthritis disease activity score,^22^ ILAR = International League of Associations for Rheumatology (ILAR) criteria ^1^ TNF = tumor necrosis factor

*Independent Samples Median Test *p* = 0.87; **Pearson chi-square *p* = 0.53

To examine longitudinal changes in serum BSSL levels, 12 of the 102 children, all with a documented positive treatment response, were included based solely on the availability of a follow-up serum sample in the biobank obtained at a visit with lower disease activity. This subgroup therefore represents a convenience sample. These children underwent a second clinical and laboratory assessment at this follow-up visit.

#### Healthy controls

A group of 20 age- and sex-matched healthy children, admitted for minor surgical procedures, served as controls (Table 1). Blood samples were collected immediately prior to surgery. Exclusion criteria included ongoing medication for any acute or chronic disease and any presence of inflammatory or autoimmune disease, diabetes, or atopic disease requiring continuous medication or a special diet for allergy or intolerance.

### Clinical assessments

At each study visit, clinical disease activity in children with JIA was assessed using the juvenile disease activity score (JADAS27) [22]. This composite index includes the active joint count (0–27 joints), the patient- or parent-reported global assessment of well-being on a 10-cm visual analog scale (VAS) (a parent served as proxy if the child was ≤ 9 years old), the physician’s global assessment of disease activity on a 10-cm VAS, and the normalized erythrocyte sedimentation rate (ESR) calculated as (ESR in mm/h − 20)/10 and scaled to 0–10. The total JADAS27 ranges from 0 to 57, with higher scores indicating greater disease activity. We also assessed the three-variable clinical cJADAS27 (0–47) which excludes the acute-phase reactant [23]. In the follow-up group we also assessed the 10-joint JADAS (JADAS10) (0–40) which includes a maximum of ten joints, to evaluate clinical improvement according to Horneff et al. [24]. In this assessment predefined cut-offs for improvement is used according to three baseline classes in the JADAS10.

Physical function was assessed using the Child Health Assessment Questionnaire (CHAQ) [25], which measures functional disability across multiple domains, with scores ranging from 0 (no disability) to 3 (severe disability).

### Blood sampling and processing

Peripheral venous blood was collected from each participant, both children with JIA and healthy controls. Samples were centrifuged at 2000xg for 10 min within 3.5 h of collection. The resulting serum aliquotes were promptly frozen at -70 °C to preserve protein stability until analysis.

### Laboratory measurements

#### Bile salt-stimulated lipase (BSSL)

Serum BSSL concentrations were measured using a Meso Scale Discovery (MSD) assay developed and qualified for human plasma by BioAgilytix (Hamburg, Germany) for Lipum AB (Umeå, Sweden). The assay uses a humanized anti-human BSSL antibody (SOL-116, Lipum AB) as the capture antibody and a polyclonal rabbit anti-human BSSL antibody (pAb7a) [26], as the detection antibody. Plates were read on the Meso QuickPlex SQ 120 platform, and data were analyzed using MSD Discovery Workbench 4.0 software. Samples were analyzed in duplicate. BSSL concentrations were calculated from a standard curve and expressed as pg/mL.

#### S100A8/A9 (Calprotectin)

Serum concentrations of the S100A8/A9 heterodimer were measured using a commercial ELISA kit (R&D Systems, Minneapolis, MN) according to the manufacturer’s instructions. Optical density was measured using a Varioskan LUX (Thermo Fisher Scientific, Waltham, MA), and data were analyzed with SkanIt Software 7.0.2 for Multiplate Readers.

#### Human neutrophil Lipocalin (HNL)

Serum HNL concentrations were measured using an ELISA (Diagnostics Development, Uppsala, Sweden). The assay preferentially, but not exclusively, detects the dimeric form of HNL, which is released from neutrophils upon priming.

#### Routine laboratory parameters

ESR was determined according to standard procedures at the Department of Clinical Chemistry and Pharmacology, Uppsala University Hospital, Uppsala, and normalized for use in JADAS27. Leukocyte, neutrophil, and platelet counts were obtained using automated cell counters at the same department.

### Statistical analyses

All statistical analyses and figure generation were performed using GraphPad Prism version 10 (GraphPad Software, La Jolla, CA). Correlations between BSSL concentrations and clinical or laboratory variables were evaluated using Spearman’s rank correlation (ρ). Differences between two independent groups were analyzed using the Mann-Whitney U test. For comparisons among multiple groups, the Kruskal-Wallis exact test was applied, followed by Dunn’s multiple-comparison tests (JIA categories versus healthy controls) when a significant overall difference was detected. Discriminative performance of serum BSSL for JIA versus healthy controls was evaluated using receiver operating characteristic (ROC) curve analysis, with calculation of the area under the curve (AUC). The optimal cut-off value was defined as the threshold that maximized the Youden index (sensitivity + specificity − 1). Longitudinal (paired) changes in BSSL levels before and after treatment in the subset of 12 children with JIA were analyzed using the Wilcoxon signed-rank test. Data are presented as median [interquartile (IQR)], unless otherwise stated. All tests were two-sided, and a p-value < 0.05 was considered statistically significant.

## Results

### Patient characteristics

A total of 102 patients with JIA were included in the study. More than half were classified within the oligoarticular JIA category, and over 90% of the whole cohort were either newly diagnosed or experiencing a disease flare at the time of enrolment. Consequently, few patients (*n* = 9, 8.8%) were treated with methotrexate or prednisolone upon inclusion. Table 1 summarizes the demographic and clinical characteristics of the study population.

### Cross-sectional analysis of BSSL in JIA versus healthy controls

**Fig. 1:**
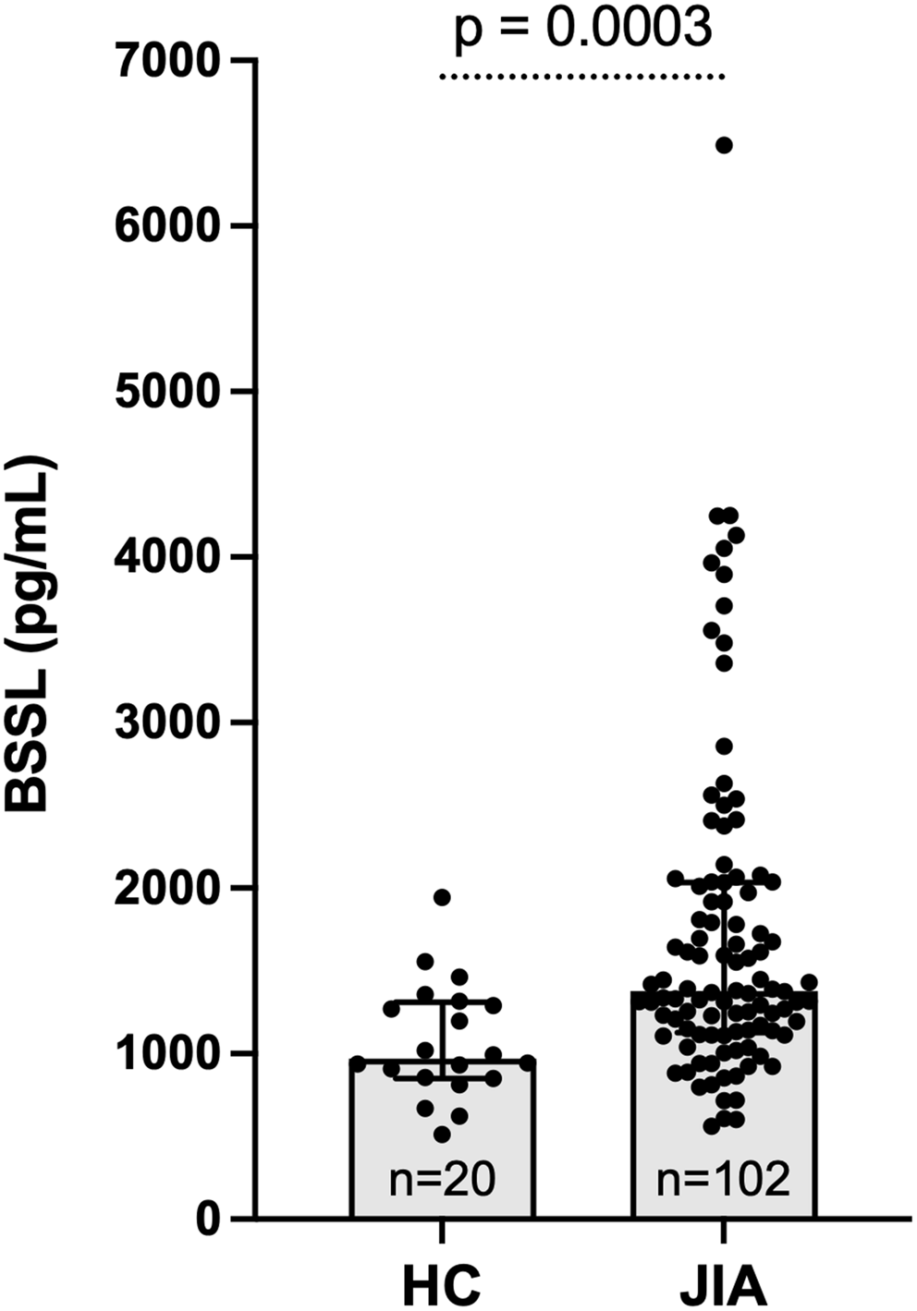
Serum BSSL concentrations in children with JIA (*n* = 102; all with active inflammatory disease), compared with healthy age- and sex-matched controls (*n* = 20). Bars represent median (IQR); differences were tested using the Mann-Whitney U test, *p* = 0.0003

Children with JIA demonstrated significantly higher serum BSSL concentrations compared to healthy children (Fig. 1). Median (IQR) BSSL concentration was 1378 (1128–2034) pg/mL in JIA (*n* = 102) versus 969 (852–1311) pg/mL in controls (*n* = 20). The Hodges-Lehmann median difference (JIA – control) was 411 pg/mL (exact 95.06%, CI 196–678). Mann-Whitney U test, two-sided *p* = 0.0003.

Receiver operating characteristic (ROC) analysis showed that serum BSSL concentrations discriminated children with JIA from healthy controls with an AUC of 0.75 (95% CI 0.64–0.86). At the cut-off maximizing the Youden index (approximately 1030 pg/mL), sensitivity was 82% and specificity was 60%, indicating moderate discrimination with substantial overlap between groups.

### Correlations between serum BSSL concentrations and disease activity markers

Spearman’s rank correlation analyses showed that serum BSSL concentrations in children with active JIA (*n* = 102) correlated most significantly with platelet counts (ρ = 0.568, *p* < 0.0001) and ESR (ρ = 0.520, *p* < 0.0001). Significant positive correlations were also observed with S100A8/A9 (ρ = 0.427, *p* < 0.0001), leukocyte counts (ρ = 0.303, *p* = 0.0020), and neutrophil counts (ρ = 0.308, *p* = 0.0042) (Table 2). Weaker associations were also seen with HNL (ρ = 0.231, *p* = 0.028) and CHAQ (ρ = 0.253, *p* = 0.0103). The correlations with JADAS27 or cJADAS27 did not reach significance (ρ = 0.195, *p* = 0.0516) and (ρ = 0.164, *p* = 0.099) respectively; however, analysis of the separate variables included in JADAS27 revealed a weak positive correlation between physician’s global assessment of disease activity and BSSL levels (ρ = 0.247, *p* = 0.012).

**Table 2:** Correlations between serum BSSL concentrations and disease associated variables in 102 children with juvenile idiopathic arthritis

Variable	Correlation coefficient	*P*-value*		Samples (*n*)
ESR mm/h	0.520	< 0.0001	****	100
Leukocytes 10^9^/L	0.303	0.0020	**	102
Neutrophils 10^9^/L	0.308	0.0042	**	85
Platelets 10^9^/L	0.568	< 0.0001	****	99
S100A8/A9 µg/L	0.427	< 0.0001	****	101
HNL µg/L	0.231	0.028	*	91
CHAQ (0–3)	0.253	0.0103	*	102
JADAS 27 (0–57) cJADAS27 (0–47)	0.195 0.164	0.0516 0.099	n.s. n.s.	100 102
PPGA	0.160	0.109	n.s.	102
PGA	0.247	0.012	*	102
Active joints	0.039	0.695	n.s.	102

* Spearman’s rank order correlation

Abbreviations: BSSL = bile salt-stimulated lipase, ESR = erythrocyte sedimentation rate, HNL= human neutrophil lipocalin, CHAQ = child health assessment questionnaire,^25^ JADAS27 = juvenile arthritis disease activity score ^22,^ cJADAS27 = clinical JADAS27^23^, PPGA = parent’s/patient’s assessment of child’s well-being, PGA = physician’s global assessment of disease activity

### Longitudinal changes in BSSL following treatment

To examine whether BSSL levels change with therapy, a subset of 12 children with JIA was followed longitudinally. Serum BSSL concentrations were assessed at two time points: first at study inclusion during active disease when all patients were untreated, and subsequently at a follow-up visit after (or during) pharmacologic intervention, at which time patients had exhibited a clinical response characterized by reduced disease activity, defined according to Horneff et al. [24]. Demographic data for these 12 children are presented in Table 3. The median (IQR) baseline BSSL level in the subgroup of 12 children with follow-up samples was 1660 (1123–3235) pg/mL, compared with 1372 (1132–1980) pg/mL in the remaining cohort (*n* = 90), with no statistically significant difference between groups (Mann–Whitney U test, *p* = 0.35).

**Fig. 2:**
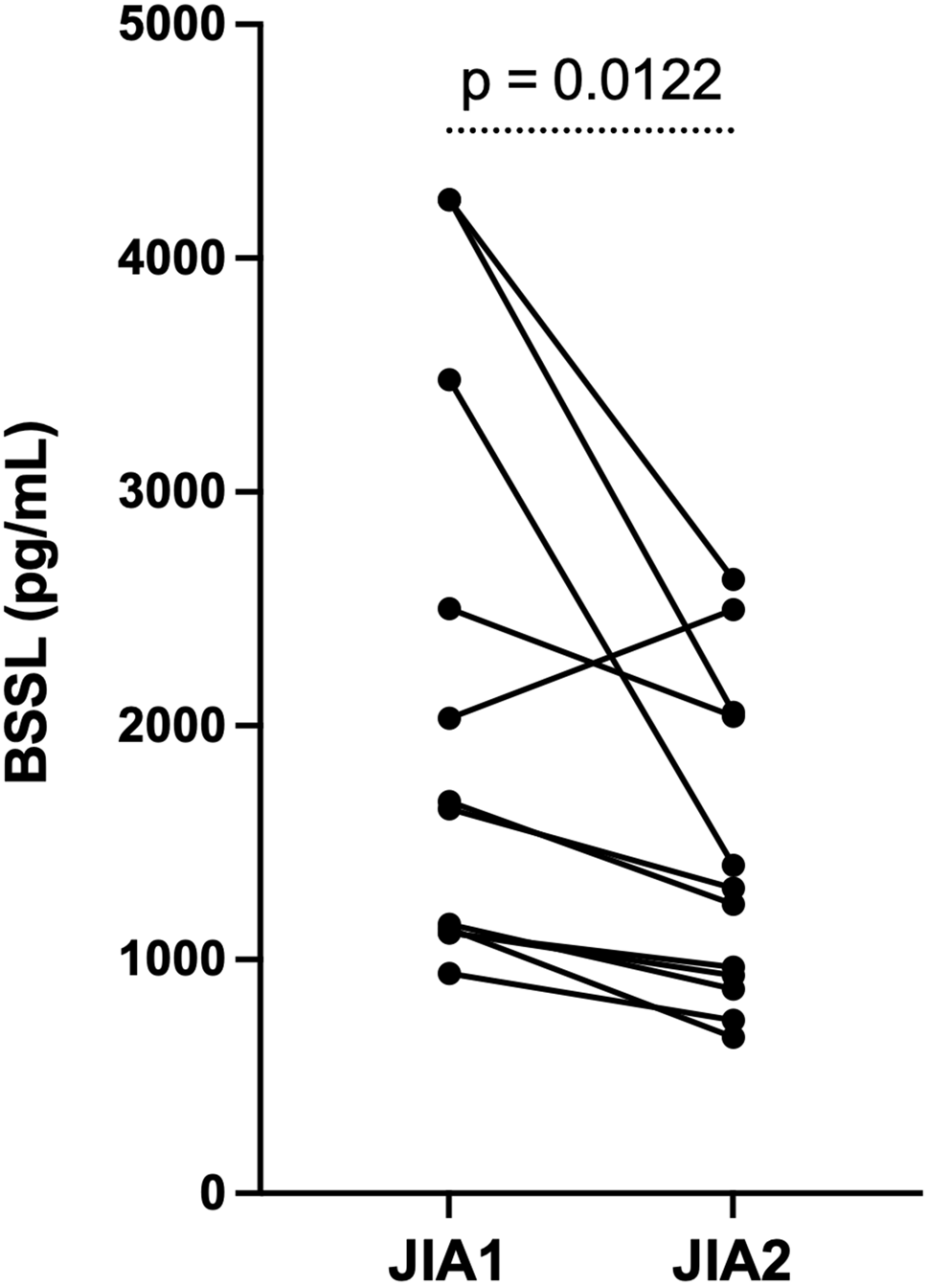
Individual serum BSSL trajectories for 12 children with JIA sampled at high and lower disease activity after initiating therapy. Lines connect paired samples from the same child. Differences were tested with the Wilcoxon signed-rank test, *p* = 0.0122. JIA1 = sample at high disease activity; JIA2 = sample at low disease activity

In paired samples, median (IQR) serum BSSL decreased from 1660 (1123–3235) pg/mL at baseline to 1271 (889–2053) pg/mL after treatment (Fig. 2). The median paired difference (baseline – post-therapy) was 390 pg/mL with an exact (96.14%) CI of 183 to 1623; Wilcoxon signed-rank test (two-sided), *p* = 0.012.

**Table 3:** Demographic data at inclusion and at follow-up in 12 children with juvenile idiopathic arthritis (JIA)

Participant		At inclusion				At follow-up		
	Gender	Age, years	JIA category	JADAS27	Medication	JADAS27	Medication	Duration, years^b^
A	F	7.7	Polyarticular RF neg	10.6	0	0	0	1.8
B	F	15.0	Polyarticular RF neg	19.9	0	3.1	Etanercept	1.6
C	F	3.6	Oligoarticular pers.	22.2	0	0	Adalimumab	4.3
D	M	8.0	Oligoarticular pers.	8.7	0	0	Mtx	1.5
E	M	13.8	Oligoarticular pers.	11.8	0	7.5	0	0.8
F	F	11.7	Polyarticular RF neg	8.6	0	0	Mtx	0.6
G	F	13.5	Polyarticular RF pos	16.9	0	0	Etanercept	1.5
H	F	14.5	Polyarticular RF pos	27.1 (16.1)^a^	0	missing	Etanercept + Mtx	0.7
I	F	12.8	Juvenile psoriatic arthritis	12.2	0	6.3	Etanercept + Mtx	0.4
J	F	15.1	Oligoarticular pers.	11.7	0	0.1	0	2.0
K	M	11.3	ERA	14.8	0	0	Mtx	0.4
L	F	15.9	Polyarticular RF pos	21.0 (18.0)^a^	0	4.5	Etanercept + Mtx	0.8

Abbreviation: ILAR = JIA category according to the International League of Associations for Rheumatology criteria^1^, JADAS27 = Juvenile Arthritis Disease Activity Score 27^22^; F = female, M = male, RF = rheumatoid factor, neg = negative, pos = positive, ERA = enthesitis-related arthritis, pers. = persistent, Mtx = methotrexate; Toc. = tocilizumab

^a^JADAS10^24^

^b^Disease duration between sampling

### BSSL levels across JIA categories

**Fig. 3:**
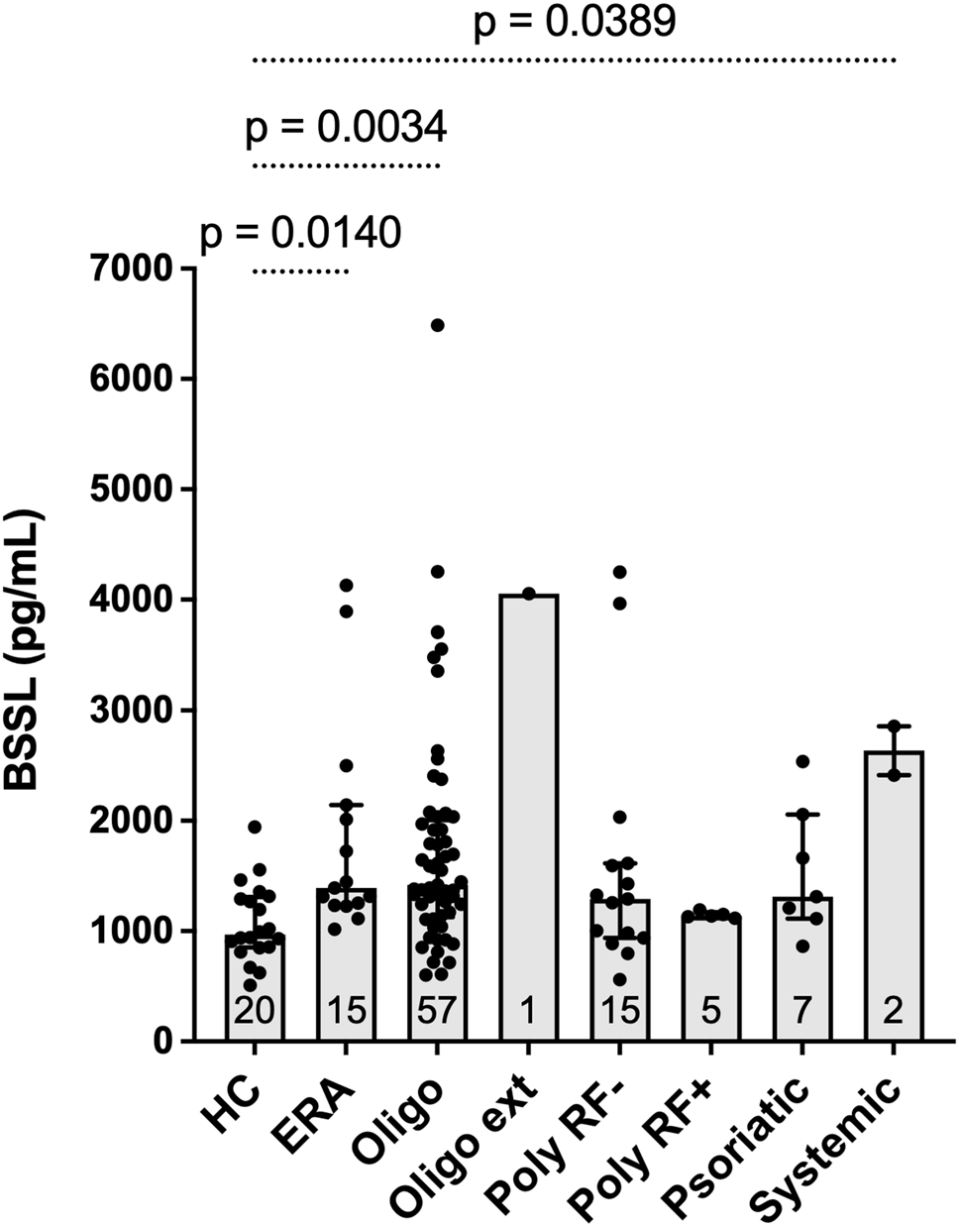
Serum BSSL concentrations by ILAR categories at study entry (active JIA) versus healthy control children. Bars represent median (IQR); numbers at the base of each bar indicate the number of subjects per group. Group differences were analyzed using the Kruskal-Wallis test followed by Dunn’s multiple-comparison test

Serum BSSL concentrations differed significally across ILAR categories (Kruskal-Wallis, H = 22.40; *p* = 0.0022). Compared with healthy controls, concentrations were higher in oligoarticular JIA, enthesitis-related arthritis, and systemic JIA (although only two participants were classified with systemic JIA) (Fig. 3; Table 4). The median levels of BSSL were higher also in the juvenile psoriatic JIA, RF-negative polyarticular JIA, and RF-positive polyarticular JIA but did not differ significantly from controls after Dunn’s multiple-comparison adjustment. Pairwise comparisons between JIA categories did not reveal any statistically significant differences in serum BSSL concentrations. These category-specific findings should be considered exploratory due to uneven sample sizes, particularly for systemic JIA (*n* = 2) and RF-positive polyarticular JIA (*n* = 5).

**Table 4:** Serum BSSL by JIA category versus healthy controls

Category	*n*	Median [IQR] pg/mL	HL diff vs. HC pg/mL	Exact rank‑based CI (achieved %)	Dunn‑adjusted *p*
Oligoarticular persistent	57	1419 [1141–1972]	448.5	198–726 (95.02%)	0.0034
Polyarticular RF negative	15	1293 [962–1604]	260.0	-37–600 (95.37%)	0.6285
Enthesitis-related arthritis	15	1391 [1243–2078]	459.5	208–874 (95.37%)	0.0140
Polyarticular RF positive	5	1139 [1132–1151]	175.0	-185–321 (95.77%)	0.9999
Juvenile psoriatic arthritis	7	1314 [1162–1860]	370.5	-3–860 (95.22%)	0.4939
Systemic	2	2636 [2525–2747]	1561.0	915–2188 (96.54%)	0.0389
Oligoarticular extended	1	4054 [4054–4054]		—	0.1628

Abbreviations: IQR = interquartile range; HL = Hodges–Lehmann, CI = confidence interval, HC = healthy controls, RF = rheumatoid factor

Values are median [IQR]. Hodges–Lehmann (HL) location shift is Category − Control; exact rank‑based confidence intervals are shown with achieved coverage in parentheses. P‑values are Dunn‑adjusted (Kruskal–Wallis with Dunn’s multiple comparisons, two‑sided). Oligo‑extended is descriptive (*n* = 1)

## Discussion

In this study, we demonstrated that serum BSSL concentrations are elevated in children with JIA, particularly during active disease, and decrease significantly following effective medical treatment with decreased disease activity. These findings are consistent with observations in adults with RA, in whom BSSL levels declined after infliximab therapy [17]. Collectively, our data and previous reports suggest that BSSL is involved in the inflammatory cascade that underlies both adult and pediatric inflammatory arthritis.

Our results further show significant correlations between BSSL concentrations and established biomarkers of systemic inflammation, including ESR, S100A8/A9, and platelet counts. These associations support the notion that BSSL reflects the degree of systemic inflammation in JIA. By contrast, BSSL did not correlate significantly with the JADAS27 or cJADAS27 composite scores. This discrepancy may reflect the multi-dimensional nature of JADAS27, which integrates joint counts, patient/parent global assessments, and physician global assessments in addition to normalized inflammatory markers [22]. It is of note however, that there was a positive correlation between physician’s global assessment of disease activity and BSSL levels. Consistent with this interpretation, ROC analysis demonstrated only moderate discrimination between children with JIA and healthy controls, with substantial overlap between groups, indicating that BSSL is unlikely to function as a stand-alone diagnostic marker but rather reflects systemic inflammatory activity. Previous research has emphasized the role of innate immunity, particularly neutrophils, in driving inflammatory joint disease, including JIA [7, 27]. Platelets, long recognized as markers of disease activity in JIA [28], also appear to play an active role in pathogenesis by forming aggregates with pro-inflammatory neutrophils [29, 30]. BSSL, which has been shown to be stored in platelets [20], may be released during platelet activation, thereby linking BSSL to the broader inflammatory milieu. This connection could explain the correlation observed between BSSL and platelet counts, suggesting that BSSL release may be a feature of platelet activation in JIA.

Interestingly, we found that BSSL levels were significantly elevated in oligoarticular, enthesitis-related, and (in a very limited number of cases) systemic JIA, but not in RF-positive polyarticular JIA, which is the JIA category that most closely resembles adult RA. Although the small sample size of the RF-positive group (*n* = 5) precludes firm conclusions, it is noteworthy that elevated BSSL has been reported in adult RA cohorts [17]. However, exploratory comparisons between JIA categories did not identify any category with significantly higher serum BSSL levels than others. Larger studies are warranted to determine whether these apparent differences represent true biological variation or are attributable to sample size limitations. In future work, it would be interesting to explore whether BSSL shares associations with joint–gut immune interactions, particularly in the ERA category but also in other JIA categories [31, 32].

Mechanistically, BSSL deficiency has been shown to confer protection against arthritis in rodent models, suggesting that BSSL can potentiate inflammatory pathways [21]. It has been hypothesized that BSSL facilitates monocyte and neutrophil recruitment or activation at inflammatory sites, thereby amplifying tissue injury and clinical manifestations [17, 21]. The significant reduction in BSSL concentration following successful treatment in our JIA cohort reinforces the likelihood that controlling BSSL levels may form part of a broader anti-inflammatory strategy. Indeed, therapies targeting BSSL, such as the humanized anti-BSSL antibody, SOL-116, have shown promise in early clinical studies by reducing circulating free BSSL levels without causing serious adverse events (ClinicalTrials.gov NCT05576012).

While our findings provide novel insights, the present study has several limitations. First, the primarily cross-sectional design (with a small longitudinal subset) precludes causal inference. Second, some JIA categories (e.g., RF-positive polyarticular) were underrepresented, limiting generalizability across all JIA subtypes. Third, although BSSL correlated significantly with markers of inflammation, it did not correlate with the composite JADAS27 or the cJADAS27 scores, warranting further exploration of disease-specific factors and differential immune pathways.

Looking ahead, larger prospective studies including all JIA categories could better define BSSL’s diagnostic or prognostic utility. BSSL is stable in both serum and plasma, which is advantageous for clinical applications, and may serve as a novel biomarker for JIA. Mechanistic studies, ideally employing ex vivo stimulation assays and tissue-specific analyses, are needed to determine whether BSSL actively drives inflammation or primarily reflects ongoing inflammatory processes. Finally, therapeutic trials targeting BSSL will help clarify its potential as a disease-modifying agent in JIA, particularly among patients who are refractory to current biologic or small-molecule therapies.

## Conclusions

This study supports a role for BSSL as a novel inflammatory biomarker in JIA. Serum BSSL concentrations were significantly elevated in children with JIA compared to healthy controls and declined following effective medical treatment, indicating responsiveness to changes in systemic inflammatory activity. BSSL correlated significantly with established inflammatory markers, including platelet counts, ESR, and S100A8/A9, confirming its association with systemic inflammation. Collectively, these findings identify BSSL as a potential biomarker for disease activity and treatment response in JIA and extends the immunological relevance of BSSL beyond adult RA to pediatric inflammatory disease. Although therapeutic targeting of BSSL is not directly addressed in the present study, existing preclinical and early clinical data suggest that BSSL may represent a future therapeutic target, warranting further investigation.

## Data Availability

All data generated or analysed during this study are included in this published article.

## Abbreviations

BSSL: Bile salt-stimulated lipase
CHAQ: Child health assessment questionnaire
cJADAS27: The clinical JADAS 27-joint Juvenile Arthritis Disease Activity Score 27
HNL: Human neutrophil lipocalin
JADAS27: The JADAS 27-joint Clinical Juvenile Arthritis Disease Activity Score 27
JIA: Juvenile idiopathic arthritis
RA: Rheumatoid arthritis
